# Interventions to increase follow-up of abnormal cervical cancer screening results: A systematic literature review and meta-analysis

**DOI:** 10.1371/journal.pone.0291931

**Published:** 2024-02-21

**Authors:** Melissa Lopez Varon, Yimin Geng, Bryan M. Fellman, Catherine Troisi, Maria E. Fernandez, Ruosha Li, Belinda Reininger, Kathleen M. Schmeler, Emma Allanson

**Affiliations:** 1 Gynecologic Oncology & Reproductive Medicine, The University of Texas MD Anderson Cancer Center, Houston, Texas, United States of America; 2 Health Promotion & Behavioral Sciences, The University of Texas Health Science Center at Houston School of Public Health, Houston, Texas, United States of America; 3 Research Medical Library, The University of Texas MD Anderson Cancer Center, Houston, Texas, United States of America; 4 Biostatistics, The University of Texas MD Anderson Cancer Center, Houston, Texas, United States of America; 5 Management, Policy & Community Health, The University of Texas Health Science Center at Houston School of Public Health, Houston, Texas, United States of America; 6 Biostatistics, The University of Texas Health Science Center at Houston School of Public Health, Houston, Texas, United States of America; 7 Health Promotion & Behavioral Sciences, The University of Texas Health Science Center at Houston School of Public Health, Brownsville Regional Campus, Brownsville, Texas, United States of America; 8 The Division of Obstetrics and Gynaecology, The University of Western Australia, Perth, Western Australia, Australia; University of Leicester, UNITED KINGDOM

## Abstract

**Introduction:**

Ensuring timely follow-up of abnormal screening results is essential for eliminating cervical cancer.

**Objective:**

The purpose of the study was to review single and multicomponent interventions designed to improve follow-up of women with abnormal cervical cancer screening results. We report on effectiveness across studies, and describe what aspects of these interventions might be more impactful.

**Methods:**

Publications were searched between January 2000 and December 2022. The search included observational, quasi-experimental (pre-post studies) and randomized controlled studies describing at least one intervention to increase follow-up of women with abnormal cervical cancer screening results. Outcomes of studies included completion of any follow-up (i.e., attending a follow-up appointment), timely diagnosis (i.e., colposcopy results within 90 days of screening) and time to diagnostic resolution (i.e., days between screening and final diagnosis). We assessed risk of bias for observational and quasi-experimental studies using the Newcastle-Ottawa Scale (NOS) tool and the Cochrane collaboration tool for randomized studies. We conducted a meta-analysis using studies where data were provided to estimate a summary average effect of the interventions on follow-up of patients and to identify characteristics of studies associated with an increased effectiveness of interventions. We extracted the comparison and intervention proportions of women with follow-up before and after the intervention (control and intervention) and plotted the odds ratios (ORs) of completing follow-up along with the 95% confidence intervals (CIs) using forest plots for the interventions vs. controls when data were available.

**Findings:**

From 7,457 identified studies, 28 met the inclusion criteria. Eleven (39%) of the included studies had used a randomized design. Most studies (63%) assessed completion of any follow-up visit as the primary outcome, whereas others measured time to definite diagnosis (15%) or diagnostic resolution (22%). Navigation was used as a type of intervention in 63% of the included studies. Most interventions utilized behavioral approaches to improve outcomes. The overall estimate of the OR for completion of follow-up for all interventions was 1.81 (1.36–2.42). The highest impact was for programs using more than one approach (multicomponent interventions) to improve outcomes with OR = 3.01 (2.03–4.46), compared with studies with single intervention approaches with OR = 1.56 (1.14–2.14). No statistical risks were noted from publication bias or small-study effects in the studies reviewed.

**Conclusion:**

Our findings revealed large heterogeneity in how follow-up of abnormal cervical cancer screening results was defined. Our results suggest that multicomponent interventions were more effective than single component interventions and should be used to improve follow-up after abnormal cervical cancer screening results. Navigation appears to be an important tool for improving follow-up. We also provide recommendations for future studies and implications for policy in terms of better defining outcomes for these interventions.

## Introduction

### Rationale

The World Health Organization (WHO) cervical cancer strategy to eliminate cancer as a public health problem, calls for 70% of women to be screened with a high-performance test at least twice in their lifetime [1, 2]. In the U.S. there are currently no indicators in the Healthy People 2030 for increasing rates of follow-up of women with abnormal cervical screening results [3]. Furthermore, cervical cancer screening rates have increased over the last two decades [4], and it is estimated that 81.1% of eligible women of all races currently have access to screening [5]. The trends for cervical cancer incidence and mortality have, however, remained stagnant and improved by only 28% during the same timeframe, with 9.7 new cases per 100,000 women in 1999 and 7.5 new cases per 100,000 women in 2017 [6], with new cases disproportionately affecting women living in under-resourced communities, such as Black and Hispanic women. In order to achieve the elimination of cervical cancer as a public health problem, defined by WHO as four or fewer cases of cervical cancer diagnosed per 100,000 women per year, follow-up with appropriate diagnosis and treatment of high-risk women with abnormal screening test results remains critical. The WHO goal is that 90% of these women receive appropriate follow-up and treatment as indicated [1].

There are many factors that could be taken into consideration as barriers to adequate and timely follow up after abnormal screenings. Women are lost to follow-up in systems due partially to a lack of education about cervical cancer and the importance of early detection and treatment [7, 8], system barriers such as lack of systems in place to remind patients of appointments or lack of infrastructure and training to perform diagnostics/treatment or lack of clinical resources [9–11]. These barriers should be addressed by sound programs that would ideally address many of these at once. In 2016, the US Community Preventive Services Task Force (CPSTF) recommended that multicomponent interventions be implemented to increase participation in cervical cancer screening [12]. Multicomponent interventions are defined as “… two or more interventions to reduce structural barriers.” They performed a systematic review and found that the use of multicomponent interventions demonstrated a median increase in screening participation of 6.1 percentage points when compared with no intervention. The CPSTF states that if access to follow-up treatment is provided, these interventions might improve the health of populations in need or underserved. Despite clear evidence and recommendations for using multicomponent interventions to increase screening, the taskforce did not include recommendations for interventions for timely diagnosis and follow-up once an abnormal cervical cancer screening result is returned [12]. Increasing cervical cancer screening at the population level is only effective in preventing cervical cancer if there is an effort to improve adherence rates to diagnosis, early detection, and treatment of preinvasive disease for those with abnormal screening results.

Given the importance of timely, effective follow-up and management of women with abnormal cervical cancer screening results, we searched the literature for interventions aimed to increase these outcomes. A previous report by Yabroff *et al*. [13] in 2000 described a qualitative meta-analysis of interventions tested using randomized or concurrently controlled study designs that may impact follow-up for women with abnormal screenings. In this study the interventions were classified as cognitive, behavioral, or sociologic, and the analysis showed that combining these types of intervention approaches offered no additional benefit to follow-up outcomes as compared to interventions with a single approach. In several cases the combination of strategies resulted in lower follow-up. Additionally, they found that the most effective interventions were cognitive interventions, which were defined as those with educational components, providing new information and altering misconceptions/mis-information.

We undertook an updated assessment of the literature following Yabroff’s classifications to identify the interventions that have been used recently to increase follow-up of women with abnormal cervical cancer screening results across multiple study designs and to explore their level of effectiveness in reaching outcomes. Our goal was to assess the effectiveness of single vs. multicomponent interventions developed to improve follow-up of women with abnormal cervical cancer screening results.

## Methods

### Protocol and registration

This systematic review was conducted in accordance with the Preferred Reporting Items for Systematic Reviews (PRISMA) guidelines, and the protocol was prospectively registered with PROSPERO (CRD42020189341).

### Criteria for considering studies for this review

All studies published between January 2000 and December 2022 that described interventions targeting improvements in follow-up for women with abnormal cervical cancer screening results were considered. All study designs and methodologies were included in our search including retrospective and prospective cohort studies, other observational studies, randomized, cluster randomized or non-randomized controlled trials, quantitative and qualitative method studies, secondary data analysis and meta-analysis of data. We considered all types of study designs that had a comparison group including pre- and post-assessments of medical records, studies with a described evaluation scheme, observational studies, and randomized studies to better understand the type of intervention approaches for follow-up of abnormal screening results.

Eligible studies were those with available data for abnormal cervical cancer screening results (either visual inspection with acetic acid (VIA), cytology (Pap test) or human papillomavirus (HPV) testing), and those that provided quantifiable outcome data on follow-up of women with abnormal results and described the individual components of the interventions used to improve follow-up. Studies were restricted to English language publications. Exclusion criteria included studies without comparison groups or study protocols without defined outcomes.

### Information sources and search strategy

We searched Ovid MEDLINE, Ovid Embase, Ovid PsycInfo, EBSCO CINAHL, PubMed and Wiley-Blackwell Cochrane Library databases for publications in English language from January 2000 to December 2022. Disease, screening and follow-up concepts were searched using subject headings and keywords as needed. The search terms were combined by "or" if they represented the similar concept, and by "and" if they represented different concepts. The complete search strategies are detailed in S1–S5 Tables of S1 File.

### Study selection

Two authors (MLV and EA) independently assessed the titles and abstracts of the identified studies after duplicates were removed, using Covidence software, which was used as the review management system. Full texts were then reviewed to determine if they met the inclusion criteria.

If the abstracts were conference proceedings or protocols the authors searched for contact information from authors and if available, attempted to contact the authors and co-authors. One co-author of two abstracts responded with information that the manuscript was in development and not able to be shared for inclusion.

### Data extraction

Data were extracted independently by MLV and EA. Any differences in data extraction were discussed between the two authors and resolved. Collected data points included study design, study location, setting, type of abnormal screening result, outcome definition and type of intervention; which were classified as single component (i.e. utilizing only one intervention strategy) or as multicomponent (using more than one intervention strategy) [13]. Given the frequency of patient navigation programs in cervical cancer screening, we decided to include the use of patient navigation in our data extraction categories; this was added to what we had described in the registered PROSPERO protocol. We intended to follow the PROSPERO protocol exactly as stated, however adaptations had to be made throughout the development of the review in terms of defining the control groups, as well as performing subgroup analyses. These adaptations helped improve the quality of the current study.

We classified the types of interventions using the same categories (cognitive, behavioral, sociological as well as combined interventions) used by Yabroff *et al*. [13]. Intervention types were classified as: 1) behavioral if they were designed to act on follow-up behaviors directly with cues to action; 2) cognitive if they consisted of educational activities offering new information or knowledge; and 3) sociologic if they made use of social norms or peers to increase compliance with follow-up.

These intervention types were selected and agreed upon by both reviewers as described by Yabroff’s manuscript; the same classification was also used by others to group interventions to increase cancer screening rates [14, 15].

### Assessment of risk of bias

Risk of bias was assessed using the Newcastle-Ottawa Scale [16] for observational and quasi-experimental studies and the Cochrane collaboration tool for assessing risk of bias in randomized studies [17]. Two authors (MLV and EA) performed individual assessments at the study level using the scales independently and resolved conflicts or differences by discussion to form consensus. A score of “1” was given at any category where the study provided that information and there was no concern of bias in that category, and a score of “0” was given when there was concern for bias.

### Meta-analysis and synthesis of results

A meta-analysis using random-effects model was performed to estimate a summary average effect of interventions and to identify whether single component or multicomponent interventions were associated with increased effectiveness of follow-up. When data were provided, we extracted the comparison and intervention data, calculated and plotted the odds ratios (ORs) of completing follow-up along with the 95% confidence intervals (CIs) using forest plots for the intervention vs. control groups, p-values of <0.05 were considered statistically significant. The meta-analysis was performed separately for observational and randomized controlled studies to account for methodological variability. We assessed the studies’ heterogeneity using the *I*^2^ statistic described by Higgins *et al*. [18]. The *I*^2^ statistic was used to measure whether the studies considered in the meta-analysis are estimating the same effect. *I*^2^ values of less than 25% represent low heterogeneity, whereas *I*^2^ values between 25 and 50% represent moderate heterogeneity, between 50 and 90% represent substantial heterogeneity and between 90–100% represent considerable heterogeneity. We calculated prediction intervals to estimate between-study variance.

We estimated potential publication bias (i.e., tendency to publish either positive or negative results) using funnel plots and confirmed using the Egger’s test for small-study effects. Symmetry in a funnel plot suggests that publication bias is not present. The vertical line in the funnel plot will indicate the fixed-effects summary estimate. The other lines in the plot will represent the 95% CI for a given standard error assuming no heterogeneity among studies. The possible sources for the asymmetry are selection biases (publication bias or selective outcome reporting), poor methodological quality leading to spuriously inflated effects in smaller studies, true heterogeneity, artifact, and chance.

All statistical analyses were performed using Stata/MP v17.0 (College Station, TX).

### Summary measures

We report proportions of women with follow-up in the intervention vs. the comparison group as extracted from the papers (ORs calculated using proportions from each publication when available).

The original protocol submitted had an end date of August 2020. We updated the search until December 31, 2022. In this updated search, 698 new studies were identified and screened, and one additional paper was included for data extraction. Furthermore, we updated the search once again until December 1, 2022, no additional papers were found in this updated search.

## Results

### Study selection

We assessed the titles and abstracts of the 7,457 studies, 49 duplicate studies were removed with the EndNote X9 library tool. We used the Covidence software to manage the review (Veritas Health Innovation, Melbourne, Australia. Available at www.covidence.org). After this, the reviewers removed 7,333 studies which were not considered relevant after assessing the publications’ abstracts (these included studies without interventions, basic-research studies about HPV vaccination, non-programmatic papers, etc.).

**Fig 1:**
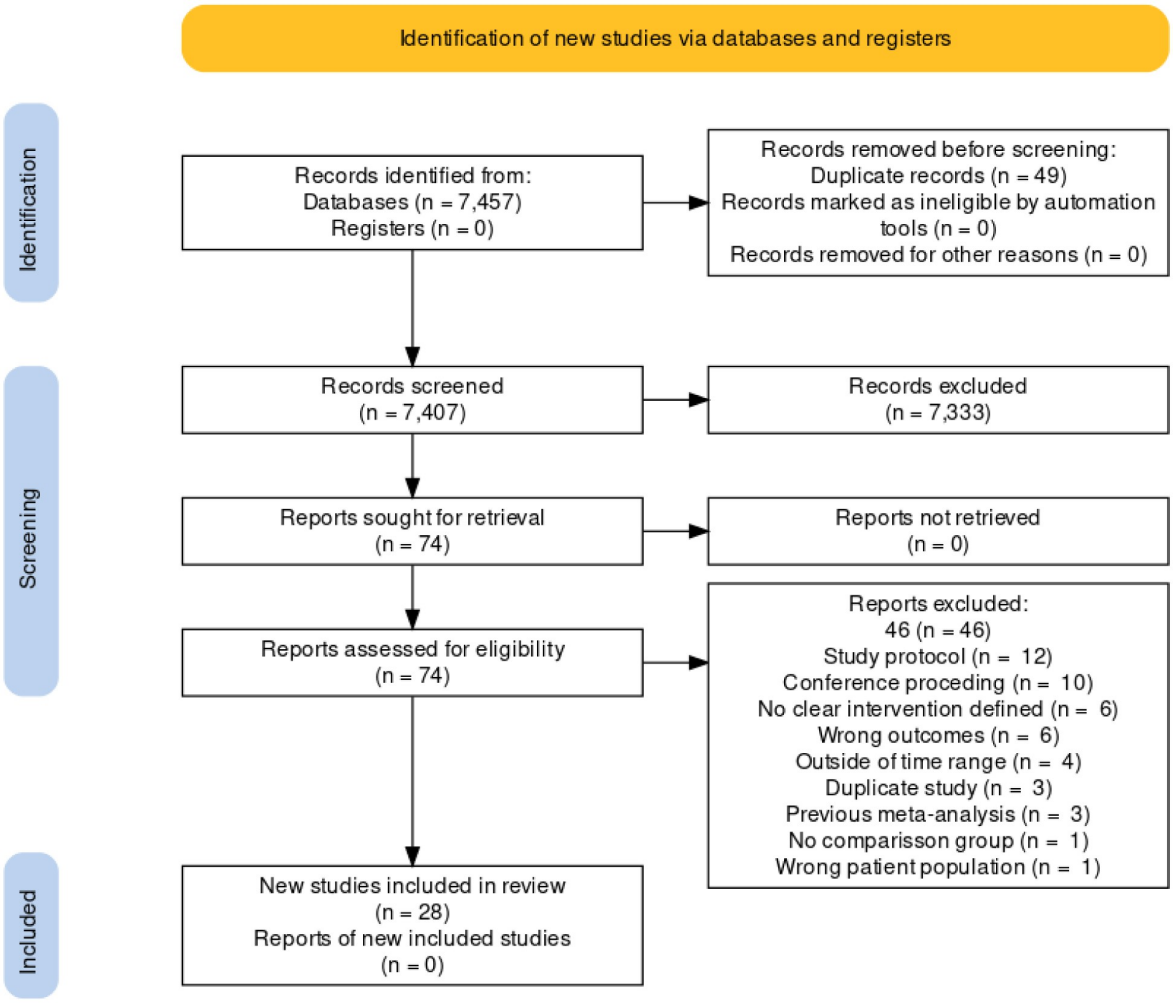
Prisma chart.

Seventy-four full text articles were reviewed, and 28 studies met the inclusion criteria **(**Fig 1**)**. Forty-six studies were removed at this step and a list of these excluded publications is available upon request.

### Characteristics of included studies

The characteristics of the 28 studies included in this review are summarized in Table 1. Of the 28 studies included, 13 were observational (cohort = 10) or quasi-experimental (pre-post = 3) studies [19–31], 11 were randomized studies [32–42] and four were a secondary data analysis: three belonging to the national patient navigation research program (PNRP), and one a meta-analysis also from the national navigation research program [43–46].

**Table 1:** Study characteristics.

	Number of studies	Percentage of studies	References
**Study design**			
Observational and quasi-experimental	13	46%	[19–31]
Randomized controlled trial	11	39%	[32–42]
Secondary analysis of data	4	14%	[43–46]
**Study location**			
United States	22	79%	[19–21, 23, 24, 26–28, 30–35, 38–40, 42–46]
Outside of the United States ^a^	6	21%	[22, 25, 29, 36, 37, 41]
**Type of patient population**			
Low-Income populations	10	36%	[20, 22, 23, 26, 31, 34, 35, 42, 46, 47]
Racial Minorities	5	18%	[21, 24, 30, 40, 43]
Women older than 65 years of age	1	4%	[33]
Combination	3	11%	[28, 32, 45]
Not stated	9	32%	[19, 25, 27, 29, 36, 37, 41, 44, 45]
**Type of abnormal exam**			
Citology	23	82%	[19–25, 27, 28, 30–32, 34, 35, 38, 39, 41–46]
HPV Test	2	7%	[37, 40]
VIA/VILI	1	4%	[29]
Not specified	2	7%	[26, 33]
**Main outcome measurement**			
Completion of any follow up visit	18	64%	[20–22, 24–27, 29, 30, 32, 34–37, 40–42, 47]
Diagnostic resolution	4	14%	[19, 23, 44, 45]
Time to definite diagnosis	6	21%	[28, 31, 33, 39, 43, 46]
**Follow up adherence assessed**			
1–3 months	4	14%	[19, 28, 29, 31]
4–6 months	8	29%	[20, 22, 25, 33, 35, 39, 42, 45]
7–12 months	4	14%	[23, 36, 38, 46]
12+ months	10	36%	[21, 24, 26, 30, 32, 34, 37, 40, 43, 44]
Not stated	2	7%	[27, 41]
**Characteristics of Interventions**			
**Type of intervention**			
Behavioral	9	32%	[19, 20, 23, 25, 26, 29, 33, 35, 36]
Cognitive	1	4%	[40]
Sociologic	0	0%	
Combined strategies	18	64%	[21, 22, 24, 27, 28, 30–32, 34, 37–39, 41–46]
**Complexity of intervention**			
Single Intervention	23	82%	[19, 20, 22, 23, 25–27, 29–33, 36–43, 45, 46, 48]
Multi-component	5	18%	[21, 24, 28, 34, 35]
**Use of patient navigation**			
Yes	18	64%	[19, 21, 22, 24, 26–28, 31, 33–35, 39, 42–46]
No	10	36%	[20, 23, 25, 29, 32, 36–38, 40, 41]
**Intervention delivery strategy**			
App or SMS	1	4%	[37]
Letter	5	18%	[12, 25, 29, 31, 36, 41]
Phone Calls	2	7%	[12, 26, 38]
Electronic Medical Records	1	4%	[23]
Combination	19	68%	[19–22, 24, 27, 28, 30–35, 39, 42–46]
**Target for interventions**			
Family members	1	4%	[29]
Patients	25	89%	[19–22, 24–28, 30–34, 36–46]
Providers	2	7%	[12, 23, 35]
Total number of interventions	28		

^a^ Canada, Denmark, Tanzania, Uganda, United Kingdom

The majority of included studies were performed in the United States (n = 22, 79%) and six were international including locations in Canada (n = 2), Denmark (n = 1), Tanzania (n = 1), Uganda (n = 1) and the United Kingdom (n = 1) [22, 25, 29, 36, 37, 41]. Of the 18 studies that described the location of the study, all were based in urban locations.

Many of the studies focused on the follow-up of women in low-resource populations/settings. Of the 28 studies included for data extraction, 10 focused on interventions directed towards low-income populations [20, 22, 23, 26, 31, 34, 35, 38, 42, 46], five addressed follow-up for racial minorities [21, 24, 30, 40, 43], one focused on addressing follow-up of women older than 65 years of age [33] and three served a combination of minority and low-income populations [28, 32, 39]. Nine of the studies did not describe a specific patient population group [19, 25, 27, 29, 36, 37, 41, 44, 45].

The majority of the studies focused on addressing follow-up for women with abnormal Pap test results (cytology) and two focused on women who had abnormal HPV testing results [37, 40]. Two did not specify the type of abnormal result [26, 33] and one focused on abnormal VIA results [29].

### Main outcome measurement

Follow-up of women with abnormal results was heterogeneously described in the included studies. Follow-up was defined in one of three ways: 1. completion of at least one follow-up visit (included scheduled, attended, and completed); 2. timely diagnosis (diagnostic resolution at a certain timepoint determined by the authors); and 3. time to definitive diagnosis (days between screening and a final diagnosis). Of the 28 studies, 18 (64%) measured completion of any follow-up visit (including completion of colposcopy or any follow-up appointment). Four of the 28 (14%) studies measured timely diagnostic resolution at a given time (a time between screening and diagnosis as defined by the authors) [19, 23, 44, 45] and 6 of the 28 (21%) studies focused on measuring time to definite diagnosis [28, 31, 33, 39, 43, 46].

The measured time to follow-up ranged between 1–3 months [19, 28, 29, 31], 4–6 months [20, 22, 25, 33, 35, 42, 45], 7–12 months [23, 36, 38, 46], two did not state the time of follow-up [27, 41] and 11 followed patients for 12+ months.

### Characteristics of the interventions described in the selected studies

Patient navigation was the most common type of intervention implemented in the studies we reviewed. Of the included 28 studies, 10 did not use navigation [20, 23, 25, 29, 32, 36–38, 40, 41] and 18 used navigation, including nine studies funded as part of the National PNRP supported by the National Cancer Institute (NCI) [48]. This program uses navigators to address and overcome barriers to cancer screening and follow-up. Some of the publications were from sites that participated in a nine-site national research program developed to overcome barriers in delays in cancer care, targeting populations that are more vulnerable such as Hispanic-Latina and Black populations. The intervention delivery strategies included text messages [37], letters [25, 29, 36, 40, 41], phone calls [26, 38], processes embedded within the electronic medical record [23] and combinations of multiple communication styles (n = 19). Most of the interventions addressed changes at the patient level (n = 25), one focused on educating family members [29] and two addressed barriers for medical providers [23, 35].

Of the 28 studies, nine were classified as behavioral interventions [19, 20, 23, 25, 26, 29, 33, 35, 36], one of them used a cognitive (educational) approach [40], and none used the sociologic approach as described by Yabroff [13]. The majority (n = 18, 64%) used combined permutations of the three main approaches. Details and examples of interventions are shown in Table 2.

**Table 2:** Examples of different intervention approaches classified by mechanism of action.

Types of interventions	Examples of interventions	Navigator could perform
**Behavioral**	Appointment reminders for patients Electronic medical record-based identification of patients who need follow up Sharing pathology results with patients directly instead of sending them to the doctors’ offices.	Yes
**Cognitive**	Educational flyers, flip charts, videos about high-risk HPV Letters informing patients about the need for follow up	Yes
**Sociologic**	• Videos of women who have had abnormal screenings to educate peers	NA

Most of the interventions utilized were single component interventions (n = 23) and five were multicomponent interventions [21, 24, 28, 34, 35]. An example of a single component intervention was using navigation alone. Examples of multicomponent interventions included systems to identify patients at high-risk of missing appointments (first component) combined with patient navigators to educate patients about the importance of following up after abnormal results are received, whereby the patient navigator offers to schedule appointments and sets up reminders for patients before their appointments (second component). Another example of a multicomponent intervention was use of navigators to provide surveys identifying knowledge gaps or socioeconomic barriers for patients to receive follow-up (first component), combined with colposcopy clinics scheduled after hours or on weekends to reduce barriers to access care within working hours (second component).

#### Completion of follow-up

We calculated the odds of completing follow-up in the 21 studies where data were available. Table 3 shows the summaries of the overall estimates of patients completing follow-up along with subgroup estimates by type of intervention (behavioral, cognitive, combined), complexity of interventions (single, multicomponent), and navigation (yes, no). The overall estimate of the OR of completing follow-up in the intervention group vs. control group was OR = 1.81 (95% CI, 1.36–2.42) indicating that interventions (regardless of mechanism) significantly increased follow-up in participating women. The overall heterogeneity of the interventions assessed by the *I*^*2*^ score was high (*I*^2^ = 95.75%).

**Table 3:** Overall estimates and confidence intervals for follow-up.

Parameter	N studies	Estimate (95% CI)	p-value ^	*I* ^2^
**OR-overall**	21	1.81 (1.36–2.43)	<0.001	95.75%
**OR-Multi-component**	5	3.01 (2.03–4.46)	<0.001	58.47%
**OR-Single intervention**	16	1.56 (1.14–2.14)	0.006	96.22%
**OR-Behavioral intervention**	7	1.88 (1.10–3.21)	0.021	96.90%
**OR-Cognitive Intervention**	1	0.74 (0.25–2.17)	0.580	1-study
**OR-Combined intervention**	13	1.87 (1.31–2.68)	0.001	92.98%
**OR-No navigation**	9	1.31 (0.92–1.88)	0.139	92.32%
**OR-Navigation**	12	2.32 (1.57–3.42)	<0.001	94.01%

^p-value is test of theta = 0 (i.e., OR = 1)

-Test of group differences for type of intervention; p = 0.263;

Single component vs multicomponent interventions; p = 0.011;

patient navigation; p = 0.035

The largest intervention effects were seen in programs utilizing multicomponent interventions; patients in the intervention group had three times the odds of receiving follow-up in comparison to patients in the comparison groups, OR = 3.01 (95% CI, 2.03–4.46). The heterogeneity of these studies was moderate (*I*^2^ = 58.47%). Single interventions still had a moderate significant effect on follow-up, with patients in the intervention arm having 56% of the odds of receiving follow-up in comparison to those in the control group; OR = 1.56 (95% CI, 1.14–2.14). Multicomponent studies had significantly higher completion of follow-up compared to single component studies. The test of group differences between single and multicomponent interventions was statistically significant, indicating that both groups were substantially different (p<0.05).

When examining type of intervention according to the Yabroff classifications, the one intervention classified as cognitive alone did not have a positive impact on follow-up for patients with abnormal screening results, and patients in this intervention were less likely to receive follow-up in comparison to the control groups, OR = 0.74 (95% CI, 0.25–2.17). Interventions that combined behavioral and cognitive components had a significantly higher effect on follow-up; patients in the intervention arm had 87% of the odds to have follow-up in comparison to the control groups, OR = 1.87 (95% CI, 1.31–2.68). These studies still had a high heterogeneity of effects (*I*^2^ = 92.98%). The test of group differences between behavioral, cognitive, and combined studies was non-statistically significant.

Because of the high proportion of studies using navigation as an intervention, we assessed the ORs of follow-up for interventions using navigation as a strategy and those not using navigation. Navigation interventions had a statistically significant positive effect on follow-up with patients in the navigation group having more than twice the odds of receiving follow-up in comparison to patients in the control group, OR = 2.32 (95% CI, 1.57–3.42). In contrast the interventions not using navigation had a lower effect on follow-up that was not statistically significant, OR = 1.31 (95% CI, 0.92–1.88). The statistical test to assess group differences of navigation vs. no navigation was statistically significant (p = 0.035).

**Fig 2:**
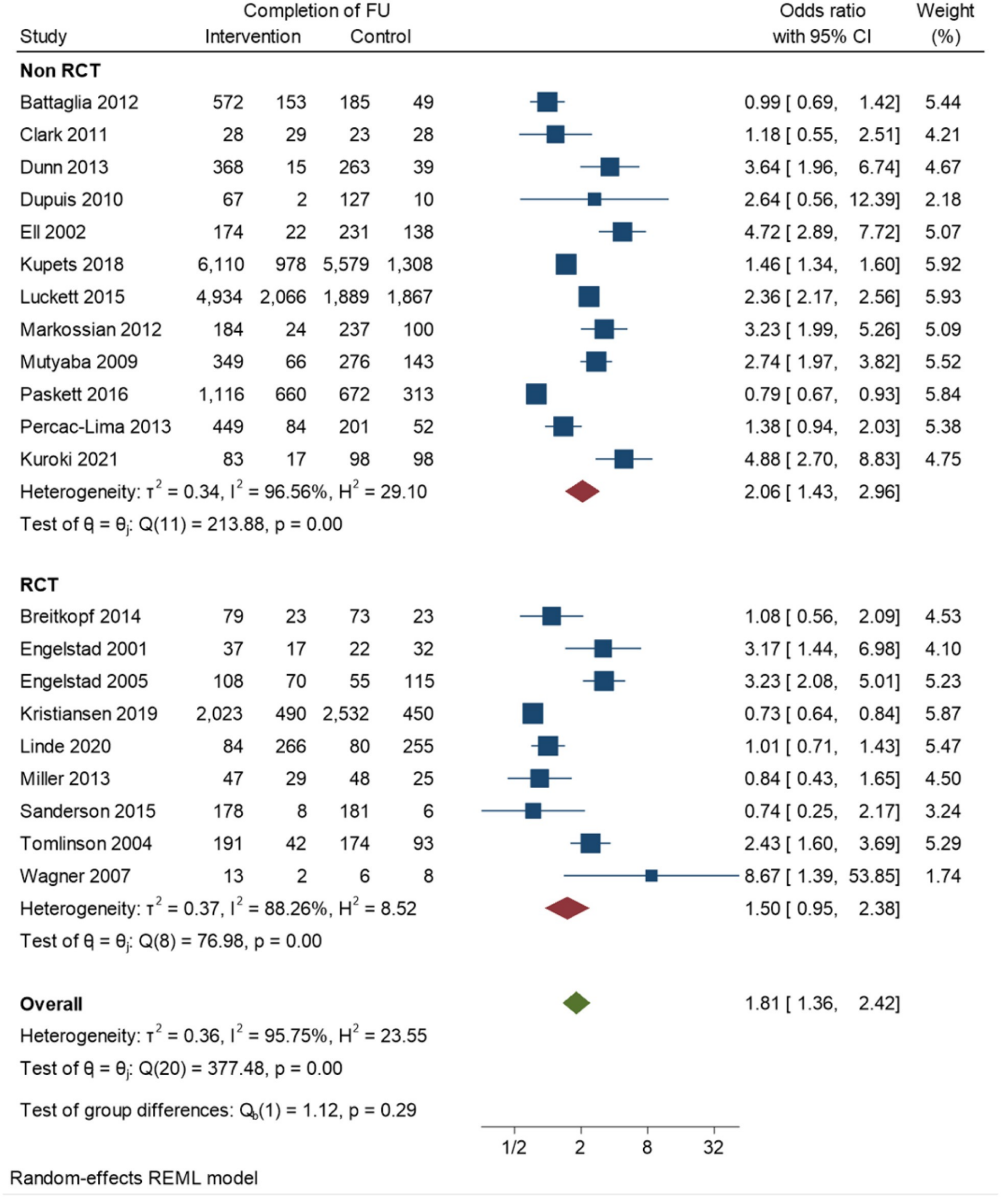
Forest plots of meta-analysis for ORs of completing follow up (RCTs and observational studies presented separately).

Fig 2 shows the forest plots of ORs for completing follow-up for the included studies. We present non-RCTs and RCTs separately. Both groups had high heterogeneity of effects, however non-RCTs had a higher heterogeneity score (*I*^*2*^ = 96.56%) in comparison to RCTs (*I*^*2*^ = 88.26%). The size and direction of the effects on both groups were statistically significant (OR = 2.06 in non-RCTs and OR = 1.50 in RCTs) indicating that the implemented interventions had an effect on the study groups regardless of the study design. The 95% prediction interval in the non-RCTs was (CI = 0.526, 8.063) and for RCTs was (CI = 0.318, 7.077).

**Fig 3:**
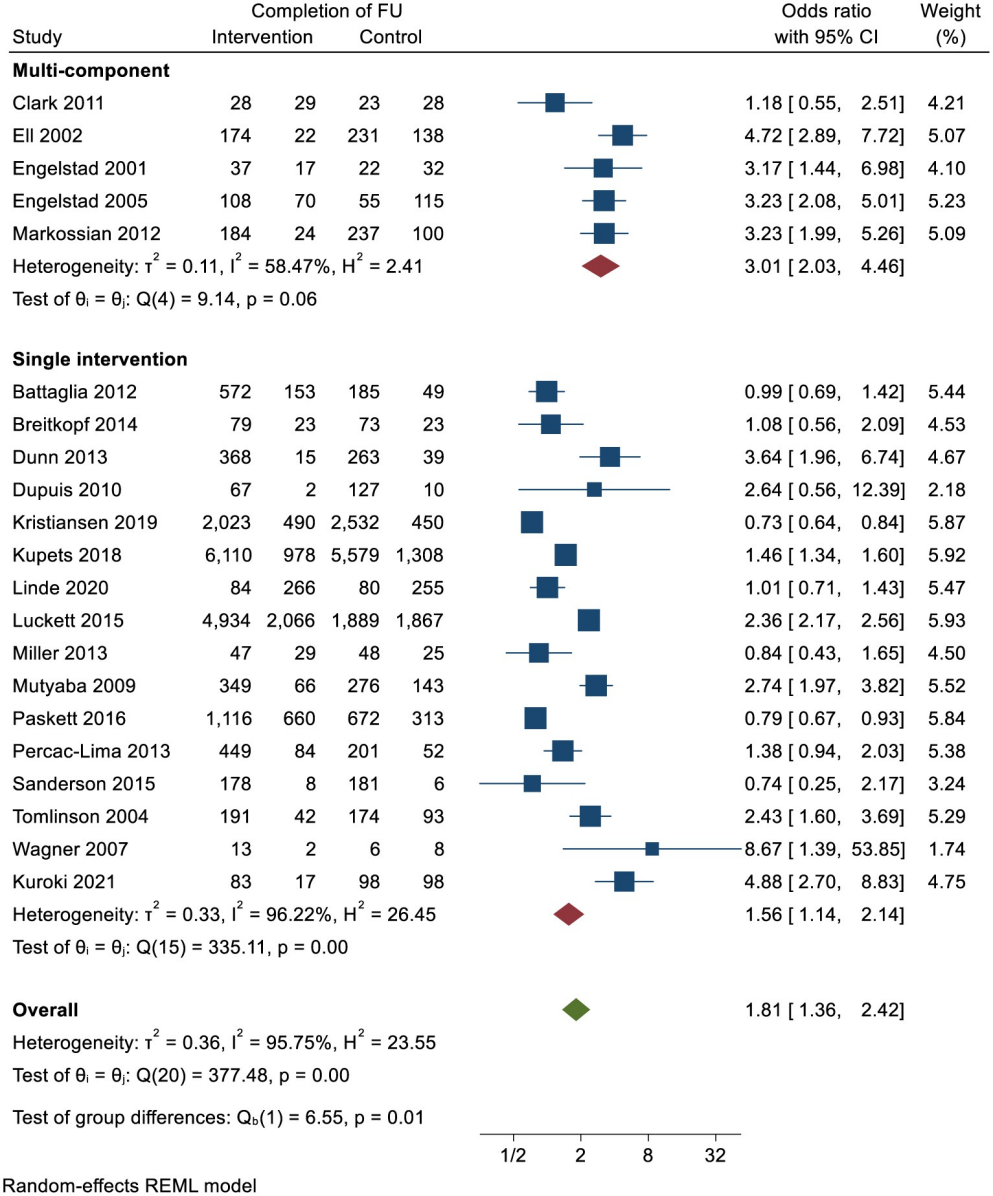
Forest plots of meta-analysis for ORs of completing follow up (single and multicomponent interventions presented separately).

As planned in our analysis we assessed completion of follow-up in the single vs. multicomponent interventions. Fig 3 shows the Forest plots for the ORs detailing the differences between multicomponent interventions and single interventions. Overall, multicomponent interventions improved completion of follow-up, OR = 3.01 (95% CI, 2.03–4.46), with moderate heterogeneity (*I*^*2*^ = 58.47%) as seen in the forest plot. By contrast, single interventions had a more modest effect on follow-up, OR = 1.56 (95% CI, 1.14–2.14), although the heterogeneity was high (*I*^*2*^ = 95.75%).

### Risk of bias assessment for quasi-experimental and cohort studies

The results of the risk of bias assessment for the 17 non-randomized before and after and cohort studies are summarized in Table 4. In terms of **selection bias**, most of the studies compared the outcomes using a representative exposed cohort, however 7 of the 17 studies (41.17%) may have some bias due to the selection of the non-exposed cohort, as these were historical controls used as baseline data prior of the intervention of the study [20, 22, 24, 26, 28, 30, 31, 45]. No bias was identified in terms of ascertainment of the exposure as this was a temporal exposure in most of the studies, likewise we did not identify concerns in the demonstration that the outcome of interest was not present at the start of the study (also due to the temporal nature of study designs). In terms of **comparability of the studies**, we identified potential bias in two of the studies where important covariates or confounding variables were either not included or not stated explicitly [24, 25]. None of the studies controlled for any additional factors in their analysis. In terms of **bias in assessment of outcome**, one of the studies was identified to have concerns about the assessment of the outcome [27]. We identified insufficient or inappropriate length of follow-up in one of the studies [24] and concerns about adequacy of follow-up in two studies [23, 24].

**Table 4:** Bias assessment for quasi-experimental and observational studies.

	SELECTION				COMPARABILITY		OUTCOME			Total
Study ID	REC	SNE	AE	DONP	SCI	SCAF	AO	LFW	FC	
Ell, 2002	**1**	**0**	**1**	**1**	**0**	**0**	**1**	**0**	**0**	**4**
Dunn, 2013	**0**	**0**	**1**	**1**	**1**	**0**	**1**	**1**	**1**	**6**
Cardini, 2001	**1**	**0**	**1**	**1**	**1**	**0**	**1**	**1**	**1**	**7**
Dupuis, 2010	**1**	**1**	**1**	**1**	**1**	**0**	**1**	**1**	**0**	**7**
Kupets, 2018	**1**	**1**	**1**	**1**	**0**	**0**	**1**	**1**	**1**	**7**
Luckett, 2015	**1**	**1**	**1**	**1**	**1**	**0**	**0**	**1**	**1**	**7**
Markossian, 2012	**1**	**0**	**1**	**1**	**1**	**0**	**1**	**1**	**1**	**7**
Mutyaba, 2009	**1**	**1**	**1**	**1**	**0**	**0**	**1**	**1**	**1**	**7**
Paskett, 2016	**1**	**0**	**1**	**1**	**1**	**0**	**1**	**1**	**1**	**7**
Percac-Lima, 2013	**1**	**0**	**1**	**1**	**1**	**0**	**1**	**1**	**1**	**7**
Simon, 2015	**1**	**0**	**1**	**1**	**1**	**0**	**1**	**1**	**1**	**7**
Charlot, 2015	**1**	**1**	**1**	**1**	**1**	**0**	**1**	**1**	**1**	**8**
Clark, 2011	**1**	**1**	**1**	**1**	**1**	**0**	**1**	**1**	**1**	**8**
Kuroki, 2021	**1**	**1**	**1**	**1**	**1**	**0**	**1**	**1**	**1**	**8**
Rodday, 2015	**1**	**1**	**1**	**1**	**1**	**0**	**1**	**1**	**1**	**8**
Battaglia, 2012	**1**	**1**	**1**	**1**	**1**	**0**	**1**	**1**	**1**	**8**
Freund, 2014	**1**	**1**	**1**	**1**	**1**	**0**	**1**	**1**	**1**	**8**

REC: Representativeness of the exposed cohort

SNE: Selection of the non-exposed cohort

AE: Ascertainment of exposure

DONP: Demonstration that the outcome of interest was not present at start of study

SCI: Study control for the most important factors (Age, SES, education, language)

SCAF: Study control for any additional factors (Mode of intervention)

AO: Assessment of outcome

LFW: Was follow-up long enough for outcomes to occur

FC: Adequacy of Follow up of cohorts

### Risk of bias assessment for randomized studies

The risk of bias for the 11 included randomized studies is summarized in Table 5. None of the studies had an overall high risk of bias and four of them had an overall low risk of bias [32, 35, 36, 38], the remaining seven studies were identified as having some concerns of risk of bias with the most common category of bias being deviations from the intended interventions [33, 34, 37, 39–41] and potential biases in the selection of the reported results. Three studies were identified as having some concerns in the randomization process.

**Table 5:** Bias assessment for randomized studies.

Unique ID	Experimental	Comparator	Outcome	D1	D2	D3	D4	D5	Overall
Breitkopf, 2014	Counseling	Standard of Care	Completion of any follow up						
Desalvo, 2018	Patient Navigation	Standard of care	Diagnostic resolution						
Engelstad, 2001	Care clinic intervention	Standard of care	Completion of any follow up						
Engelstad, 2005	Navigation	Standard of care	Completion of any follow up						
Kristiansen, 2019	Citology results from path	Standard of care	Completion of any follow up						
Linde, 2020	Text messages	Standard of care	Completion of any follow up						
Miller, 2013	Telephone reminder and counseling	Enhanced standard of care with phone reminder	Completion of any follow up						
Paskett, 2011	Patient Navigation	Standard of Care	Time to definite diagnosis						
Sanderson, 2015	Printed materials	Standard of care	Completion of follow up						
Tomlinson, 2004	Information leaflets and appt reminders	Standard of care	Completion of any follow up						
Wagner, 2007	Tailored outreach intervention	Standard of care	Completion of any follow up						

Green—Low Risk

Yellow—Some Concerns

Red—High Risk

D1: Randomization process

D2: Deviations from the intended interventions

D3: Missing outcome data

D4: Measurement of outcome

D5: Selection of the reported result

### Risk of publication bias

**Fig 4:**
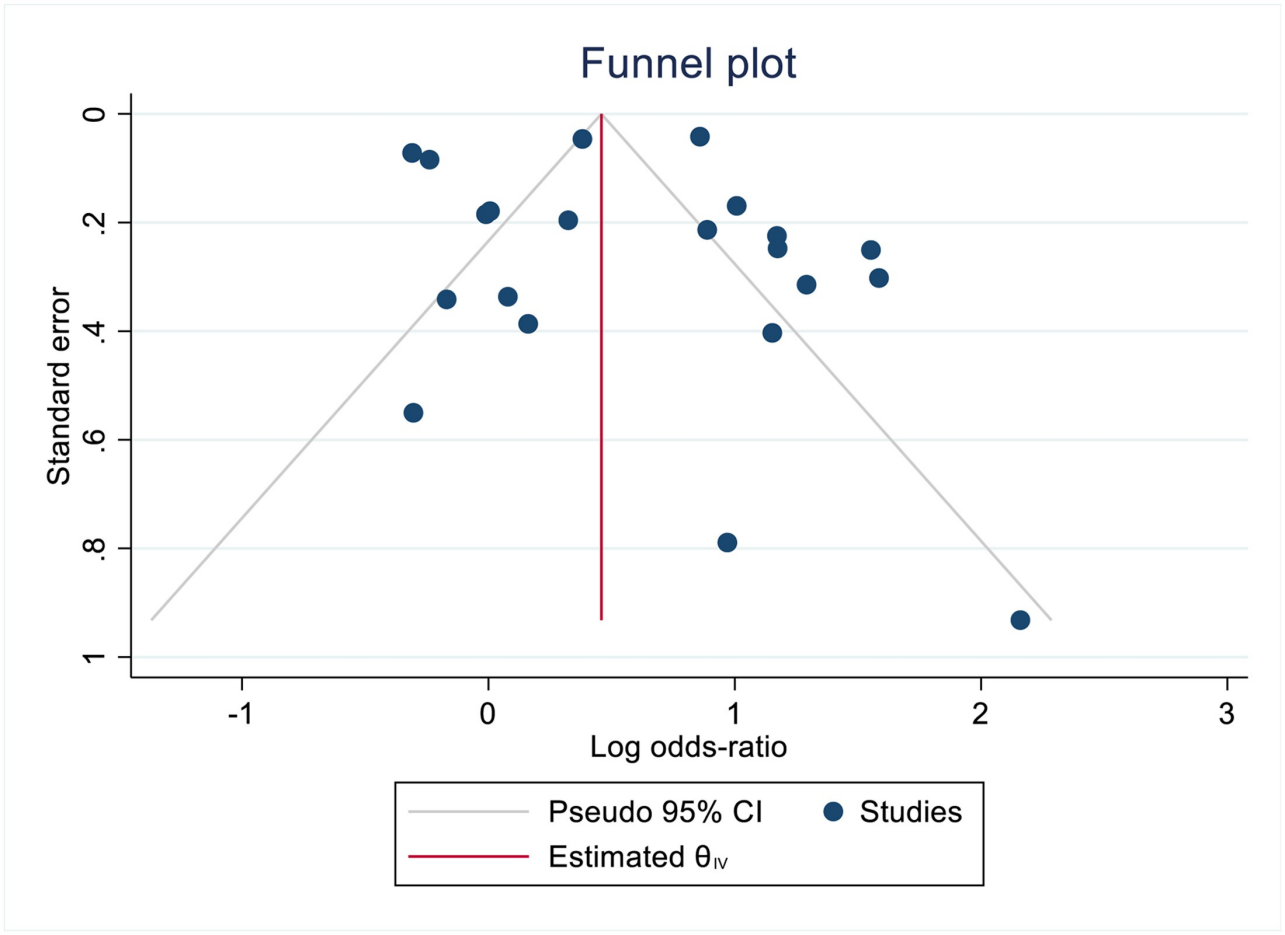
Funnel plot to identify risk of publication bias.

We assessed publication bias using a funnel plot (Fig 4), we also confirmed these results using a regression-based Egger test for small study effects. The plot in Fig 4 appears to be asymmetrical in the lower left, indicating possible bias in publication for smaller studies with negative effects, however when we confirmed using Egger’s test, the test results were Z = 1.35, greater than Z = 0.1772, which indicates that there is not a statistically significant publication bias or small-study effects in the included literature.

## Discussion

This systematic literature review gathered information from publications addressing follow-up of women with abnormal cervical cancer screening results between 2000 and 2022. A total of 7,457 papers were included in this review and we assessed the evidence of 28 studies that fit the inclusion criteria. These studies implemented either single or multicomponent interventions to improve follow-up of women with abnormal screening results. The overall OR of estimated effects was 1.81 increase in the odds of follow-up for abnormal cervical cancer screening result [95% CI, 1.36–2.42].

The greatest effect was seen with multicomponent interventions to follow-up women with abnormal results (The combined estimate of effects was OR = 1.81 higher odds of follow-up for abnormal cervical cancer screening results). This is particularly relevant and consistent with findings from systematic reviews related to screening and recommendations provided by the CPSTF recommendation to increase cervical cancer screening [12]. Our findings also suggest that multicomponent interventions should also be considered as an approach to increase follow-up for women with abnormal cervical cancer screening results.

The use of patient navigation had statistically significant effects on the follow-up of women with abnormal screening results with an OR = 2.32 (95% CI, 1.57–3.42) in navigation studies vs. OR = 1.31 (95% CI, 0.92–1.88) in studies that did not include navigation. The use of patient navigators, community healthcare workers or patient advocates has been increasing over the last decade with good outcomes for increasing cancer screening rates [21, 49–51]. Increased use of patient navigators should be considered not only in cervical cancer screening programs to increase screening rates, but also to increase patient follow-up and adherence to recommended treatment for vulnerable populations at higher risk of loss to follow-up.

When comparing types of intervention approaches as defined by Yabroff and colleagues, behavioral interventions alone (including interventions addressing/changing behaviors such as patient reminders, appointments) had an increased effect in follow-up (p = 0.021). Cognitive interventions alone had no effect on follow-up, however only one study implemented this single strategy. Interventions combining these approaches had a statistically significant effect on follow-up (OR = 1.87), emphasizing the likely importance of utilizing more than one approach for increasing success in follow-up rates.

The findings from our review differ from the results of Yabroff’s publication from 2000 [13] which concluded that multicomponent or combinations of interventions offered no additional benefits in follow-up rates. The different analytical approaches in the Yabbrof study in comparison to ours could make the direct comparison difficult.

They also concluded that the most effective interventions were cognitive interventions (as opposed to behavioral interventions) using interactive telephone counseling with an increase of compliance between 24–31%. Perhaps having someone to perform the interactive call like a “navigator” could be what made the intervention impactful. Additionally, the papers included in Yabroff’s review have similar numbers of interventions classified as cognitive and behavioral whereas in our study, most interventions were characterized as behavioral. The length of follow-up in our study was mostly classified as longer than 12 months, followed by 4–6 months. In Yabroff’s study, follow-up was mainly 4–6 months, followed by 1–3 months. Our systematic literature review included 28 studies and Yabroff’s included 10 studies. Our inclusion criteria opened the search to studies of multiple study designs whereas Yabroff’s included only controlled intervention trials with overall a shorter period of follow-up. The existing literature supports the use of multicomponent interventions to improve patient outcomes and cancer screenings [52–54], which validates our main conclusion that implementing such strategies also increases the odds of follow-up in women in the multicomponent intervention groups vs. those in the control groups.

An important strength of this study is that we included publications where the authors had used various types of study designs and outcomes defining follow-up for abnormal cervical cancer screenings globally. This broad search allowed us to include publications that used observational, quasi-experimental and experimental designs to assess the impact of their interventions. Additionally, the decision to include categories of types of interventions helped us tease out the more impactful types of interventions to improve patient follow-up. Furthermore, we performed an extensive search of the grey literature and reached out to authors of abstracts and conference proceedings seeking to learn about their findings.

This study also has limitations. The main limitation is that there is a high degree of statistical heterogeneity between the studies. There is also potential for reporting biases, particularly when multiple papers drew data from the same national patient navigation program. In terms of inclusion criteria and heterogeneity due to differences in the women included in the different studies, we consider that even though there was heterogeneity in the racial, geographic, and income characteristics of patients, the studies are addressing the needs of high-risk populations. The control groups in these studies are composed of the same populations, allowing us to be confident about the comparability of the results and that the effect of such interventions is transferable to the larger -same- populations.

One general limitation in terms of measuring outcomes is that there is currently no clear consensus on how to define appropriate follow-up of women with abnormal cervical cancer screening results. This resulted in studies providing their own definitions of follow up. We made an effort to characterize and summarize these differences by providing three categories for outcome follow up: 1. Completion of at least one follow-up appointment; 2. Diagnostic resolution; and 3. Time to definite diagnosis. Clear guidance on the definition of acceptable outcomes of follow-up for abnormal screening results is complex due to different guideline recommendations for patient follow-up depending on their individual risk. In practice, follow-up of abnormal results also needs to be paired with appropriate treatment, and this was not assessed in our study. Women with an abnormal cervical screening results are recommended to have, depending on their location and resources available [55], further diagnostic investigations and/or treatment. Diagnostic investigations include colposcopy and targeted cervical biopsy. The results of this may lead to a recommendation of treatment of confirmed high-grade cervical abnormalities, which may be done with excisional procedure (e.g., large loop excisional procedure) or cervical cryotherapy or thermal ablation. In some settings where resources are scarcer, women may proceed straight to treatment based on inspection of the cervix, without the need for colposcopy and cervical biopsy first.

Another limitation of our report is the classification of studies. We understand that this classification is not ideal as all interventions are intending to address behaviors, however we used these categories to organize the data in a meaningful way and to provide a comparable framework to update the results of the study by Yabroff *et al*. [13]. We acknowledge that there is overlap in these categories and that there is some subjectivity in how the different interventions were classified. However, to be able to organize the data in a meaningful way, and to use a framework that could be compared to a previous review, we used these three categories.

Considering that some delay in attendance, especially for high-risk populations, may be expected, some flexibility should be included as one of the considerations for success. The Centers for Disease Control and Prevention (CDC) recommends that women diagnosed with high-grade cervical lesions should be treated within 90 days [47, 56]. For clinical purposes and to align interventions, another potential measure of impact to be used, could be from the WHO’s strategy to eliminate cervical cancer as a public health problem: that 90% of women with identified abnormal results receive treatment.

Most of the studies included in this review (63%) measured completion of any follow-up, which could include attending a follow-up appointment at any time. A limitation with this type of outcome is that it does not assess the success of the interventions in terms of early diagnosis and most importantly appropriate treatment of women with preinvasive disease. Considering the current variability in definition of outcomes, our recommendation is that further studies use the framework from WHO’s elimination strategy to eliminate cervical cancer as a public health problem and use the 90% of women with abnormal results treated within a reasonable timeframe (90 days) from our clinical perspective.

Seeking to explore how these multicomponent interventions could be designed by future programs, we looked at the study with the lowest effect (Clark 2011) in this group. The authors described a need to include more efforts on implementing system changes to address the social determinants of health (including social support and childcare). In contrast, the study with the highest effect (Ell 2022) explicitly stated that their multicomponent intervention sought to address personal and systems barriers to follow-up adherence, highlighting the importance of system strengthening as part of multicomponent programs.

## Conclusions

The implementation of evidence-based interventions to improve the follow-up of women with abnormal cervical cancer screening results will aid in the goal to eliminate cervical cancer as a public health problem globally, yet this remains a challenge for populations at high risk. Our goal was to assess the effectiveness of interventions on the impact of patient follow-up after abnormal cervical cancer screening results. We conclude that even though there are challenges in describing outcomes of successful interventions for follow-up, multicomponent interventions are beneficial and impactful to improve follow-up rates. Additionally, the use of patient navigation in these interventions seems promising and should continue to be explored and assessed.

Most of the papers included in this study implemented interventions that targeted patients directly. Future interventions seeking to make improvements at the policy, system and provider level may be beneficial as there are barriers to access care at all of these levels. Future trials should also assess steps beyond adherence to an initial follow-up visit and focus on assessing the proportion of patients with appropriate treatment after receiving abnormal screening results. A target goal would be 90% of patients receiving appropriate treatment within 90 days of abnormal test results. It is important to continue to assess the cost-effectiveness of these multicomponent interventions so that they can be recommended and implemented broadly.

## Supporting information

S1 Checklist Prisma 2020 checklist for systematic reviews. (DOCX)

S1 File S1–S5 Tables. Search strategies for Ovid MEDLINE (S1), Ovid Embase (S2), Ovid PsycInfo (S3), EBSCO CINAHL (S4) and Cochrane Library (S5).
(DOCX)

S2 File Full data extracted for this systematic review with meta-analysis. (XLSX)
