# Supplementary material for: Interventions to increase follow-up of abnormal cervical cancer screening results: A systematic literature review and meta-analysis
Source: PLoS One. 2024 Feb 21;19(2):e0291931. doi: 10.1371/journal.pone.0291931 (PMC10880967; doi:10.1371/journal.pone.0291931)
Supplement: S1 File — Search strategies for Ovid MEDLINE (S1), Ovid Embase (S2), Ovid PsycInfo (S3), EBSCO CINAHL (S4) and Cochrane Library (S5). (DOCX) [file pone.0291931.s002.docx]

Supplementary Table S1. Ovid MEDLINE search strategy

| 1 | Uterine Cervical Neoplasms/ |
| --- | --- |
| 2 | Cervical Intraepithelial Neoplasia/ |
| 3 | exp Uterine Cervical Dysplasia/ |
| 4 | ((cervix or cervical) adj3 (neoplasm* or cancer or carcinoma* or precancer or precancerous or premalignant or dysplasia or neoplasia)).ti,ab,kf. |
| 5 | or/1-4 |
| 6 | limit 5 to (english language and yr="2000 -Current") |
| 7 | Mass Screening/ |
| 8 | "Early Detection of Cancer"/ |
| 9 | (screening or detection).ti,ab,kf. |
| 10 | Papanicolaou Test/ |
| 11 | ((pap or papanicolaou) adj (smear or smears or test)).ti,ab,kf. |
| 12 | (HPV adj3 (test or tests or testing)).ti,ab,kf. |
| 13 | Human Papillomavirus test*.ti,ab,kf. |
| 14 | ("human papilloma virus*" adj2 test*).ti,ab,kf. |
| 15 | Vaginal Smears/ |
| 16 | ((cervical or vaginal) adj (smear or smears)).ti,ab,kf. |
| 17 | or/7-16 [Cervical Screening] |
| 18 | 6 and 17 [Cervical Screening] |
| 19 | intervention*.ti. |
| 20 | (intervention* adj6 (clinician* or collaborat* or community or complex or DESIGN* or doctor* or physician* or practitioner* or educational or financial or GP or general practice* or hospital* or impact* or improv* or individuali?e* or individuali?ing or interdisciplin* or multicomponent or multi-component or multidisciplin* or multi-disciplin* or multifacet* or multi-facet* or multimodal* or multi-modal* or combined or personali?e* or personali?ing or prescrib* or primary care or professional* or provider* or team* or usual care or cognitive or behavioral)).ab,kf. |
| 21 | exp Patient Navigation/ |
| 22 | exp Patient Education as Topic/ |
| 23 | exp *Health Education/mt [Methods] |
| 24 | exp *health knowledge, attitudes, practice/ |
| 25 | Community Health Workers/ |
| 26 | Community-Institutional Relations/ |
| 27 | *Community Health Services/ |
| 28 | exp *Counseling/ |
| 29 | *nurses/ or nurse practitioners/ or nurses, community health/ or nurses, public health/ |
| 30 | Physician Assistants/ |
| 31 | *health personnel/ or *allied health personnel/ or health educators/ or *physicians/ |
| 32 | Physician's Role/ |
| 33 | exp Cognitive Behavioral Therapy/ |
| 34 | exp social support/ |
| 35 | Self Care/ |
| 36 | self efficacy/ |
| 37 | Self-Help Groups/ |
| 38 | ("self help" or "self efficacy").ti. |
| 39 | *"Surveys and Questionnaires"/ |
| 40 | (education* or training or navigator* or "patient navigation" or promotoras or "community health workers" or "lay health workers" or "community outreach" or "community clinic" or "federal* qualified health center*").ti. |
| 41 | (system or program or communication).ti. and intervention*.ab,kf. |
| 42 | exp Communication/ and intervention*.ab,kf. |
| 43 | nurse-patient relations/ and intervention*.ab,kf. |
| 44 | physician-patient relations/ and intervention*.ab,kf. |
| 45 | exp *Communications Media/ and intervention*.ab,kf. |
| 46 | Program Development/ and intervention*.ab,kf. |
| 47 | or/19-46 [Multi component interventions] |
| 48 | 18 and 47 [interventions after cervical screening] |

Supplementary Table S2. Ovid Embase search strategy

| 1 | exp uterine cervix cancer/ |
| --- | --- |
| 2 | exp uterine cervix dysplasia/ |
| 3 | ((cervix or cervical) adj3 (neoplasm* or cancer or carcinoma* or adenocarcinoma* or precancer or precancerous or premalignant or dysplasia or Neoplasia)).ti,ab,kw. |
| 4 | or/1-3 |
| 5 | limit 4 to (english language and yr="2000 -Current") |
| 6 | exp cancer screening/ |
| 7 | early cancer diagnosis/ |
| 8 | (screening or detection).ti,ab. |
| 9 | papanicolaou test/ |
| 10 | ((pap or papanicolaou) adj (smear or smears or test)).ti,ab. |
| 11 | (HPV adj3 (test or tests or testing)).ti,ab. |
| 12 | Human Papillomavirus test*.ti,ab. |
| 13 | ("human papilloma virus*" adj2 test*).ti,ab. |
| 14 | vagina smear/ |
| 15 | ((cervical or vaginal) adj (smear or smears)).ti,ab. |
| 16 | or/6-15 |
| 17 | 5 and 16 |
| 18 | intervention*.ti. |
| 19 | (intervention* adj6 (clinician* or collaborat* or community or complex or DESIGN* or doctor* or physician* or practitioner* or educational or financial or GP or general practice* or hospital* or impact* or improv* or individuali?e* or individuali?ing or interdisciplin* or multicomponent or multi-component or multidisciplin* or multi-disciplin* or multifacet* or multi-facet* or multimodal* or multi-modal* or combined or personali?e* or personali?ing or prescrib* or primary care or professional* or provider* or team* or usual care or cognitive or behavioral)).ti,ab. |
| 20 | exp patient education/ |
| 21 | health education/ |
| 22 | exp health promotion/ |
| 23 | Community Health Workers/ |
| 24 | preventive health service/ |
| 25 | counseling.ti. |
| 26 | exp *nurse/ |
| 27 | physician assistant/ |
| 28 | exp *health care personnel/ |
| 29 | exp health educator/ |
| 30 | exp *physician/ |
| 31 | *physician attitude/ |
| 32 | cognitive behavioral therapy/ |
| 33 | exp behavior therapy/ or exp cognitive therapy/ |
| 34 | exp interpersonal communication/ and intervention*.ab,kw. |
| 35 | exp *mass communication/ and intervention*.ab,kw. |
| 36 | (system or program or communication).ti. and intervention*.ab,kw. |
| 37 | exp *doctor patient relation/ and intervention*.ab,kw. |
| 38 | program development/ and intervention*.ab,kw. |
| 39 | *social support/ |
| 40 | exp self care/ |
| 41 | ("self help" or "self efficacy").ti. |
| 42 | exp *questionnaire/ |
| 43 | (questionnaire* or survey).ti. |
| 44 | (education* or training or navigator* or "patient navigation" or promotoras or "community health workers" or "lay health workers" or "community outreach" or "community clinic" or "federal* qualified health center*").ti. |
| 45 | or/18-44 |
| 46 | 17 and 45 |
| 47 | ((cervix or cervical) adj3 (neoplasm* or cancer or carcinoma* or adenocarcinoma* or precancer or precancerous or premalignant or dysplasia or Neoplasia)).ti,ab. |
| 48 | (cervix or cervical).ti. |
| 49 | 47 or 48 |
| 50 | 46 and 49 |

Supplementary Table S3. Ovid PsycInfo search strategy

| 1 | exp cervix/ |
| --- | --- |
| 2 | (cervix or cervical).ti,ab,id. |
| 3 | 1 or 2 |
| 4 | exp Neoplasms/ |
| 5 | (neoplasm* or cancer or carcinoma* or adenocarcinoma* or precancerous or precancer or premalignant or dysplasia or Neoplasia).ti,ab,id. |
| 6 | 4 or 5 |
| 7 | 3 and 6 |
| 8 | limit 7 to (english language and yr="2000 -Current") |
| 9 | exp Cancer Screening/ |
| 10 | (screening or detection).ti,ab,hw,id. |
| 11 | exp Human Papillomavirus/ |
| 12 | (HPV adj3 (test or tests or testing)).ti,ab,id. |
| 13 | Human Papillomavirus test*.ti,ab,id. |
| 14 | ("human papilloma virus*" adj2 test*).ti,ab,id. |
| 15 | ((pap or papanicolaou) adj (smear or smears or test)).ti,ab. |
| 16 | ((cervical or vagina*) adj (smear or smears)).ti,ab,id. |
| 17 | or/9-16 [cervical cancer screening] |
| 18 | 8 and 17 |
| 19 | intervention*.ti. |
| 20 | (intervention* adj6 (clinician* or collaborat* or community or complex or DESIGN* or doctor* or physician* or practitioner* or educational or financial or GP or general practice* or hospital* or impact* or improv* or individuali?e* or individuali?ing or interdisciplin* or multicomponent or multi-component or multidisciplin* or multi-disciplin* or multifacet* or multi-facet* or multimodal* or multi-modal* or combined or personali?e* or personali?ing or prescrib* or primary care or professional* or provider* or team* or usual care or cognitive or behavioral)).ti,ab,id. |
| 21 | exp Client Education/ |
| 22 | exp Health Education/ |
| 23 | exp Health Promotion/ |
| 24 | exp Health Personnel/ |
| 25 | exp Intervention/ |
| 26 | prevention/ |
| 27 | counseling.ti. |
| 28 | exp *Nurses/ |
| 29 | exp *Physicians/ |
| 30 | exp *Health Personnel/ |
| 31 | exp *Educational Personnel/ |
| 32 | *Training/ |
| 33 | exp cognitive behavior therapy/ |
| 34 | exp Behavior Therapy/ |
| 35 | exp Cognitive Therapy/ |
| 36 | exp Communications Media/ |
| 37 | (system or program or communication).ti. |
| 38 | exp Communication/ |
| 39 | social support/ |
| 40 | self-care skills/ |
| 41 | self-efficacy/ |
| 42 | *health behavior/ |
| 43 | questionnaires/ |
| 44 | (questionnaire* or survey).ti. |
| 45 | (education* or training or navigator* or "patient navigation" or promotoras or "community health workers" or "lay health workers" or "community outreach" or "community clinic" or "federal* qualified health center*").ti. |
| 46 | or/19-45 |
| 47 | 18 and 46 |
| 48 | ((cervix or cervical) adj3 (neoplasm* or cancer or carcinoma* or adenocarcinoma* or precancer or precancerous or premalignant or dysplasia or Neoplasia)).ti,ab,id. |
| 49 | (cervix or cervical).ti. |
| 50 | 48 or 49 |
| 51 | 47 and 50 |

Supplementary Table S4. EBSCO CINAHL search strategy

| S51 | S49 AND S50 |
| --- | --- |
| S50 | TI ( neoplasm* or cancer or carcinoma* or precancerous or precancer or premalignant or dysplasia or neoplasia or lesion* or CIN* or abnormal or positive ) OR AB ( neoplasm* or cancer or carcinoma* or precancerous or precancer or premalignant or dysplasia or neoplasia or lesion* or CIN* or abnormal or positive ) |
| S49 | S47 AND S48 |
| S48 | TI ( cervix or cervical ) OR AB ( cervix or cervical ) |
| S47 | S15 AND S45 |
| S46 | S15 AND S45 |
| S45 | S16 OR S17 OR S18 OR S19 OR S20 OR S21 OR S22 OR S23 OR S24 OR S25 OR S26 OR S27 OR S28 OR S29 OR S30 OR S31 OR S32 OR S33 OR S34 OR S35 OR S36 OR S37 OR S38 OR S39 OR S40 OR S41 OR S42 OR S43 OR S44 |
| S44 | ( (MM "Physician-Patient Relations") OR (MM "Nurse-Patient Relations") ) AND AB intervention* |
| S43 | (MM "Program Development+") AND AB intervention* |
| S42 | (MM "Communications Media+") AND AB intervention* |
| S41 | (MM "Communication+") AND AB intervention* |
| S40 | TI ( system or program or communication ) AND AB intervention* |
| S39 | TI (education* or training or navigator* or "patient navigation" or promotoras or "community health workers" or "lay health workers" or "community outreach" or "community clinic" or "federal* qualified health center*") |
| S38 | (MM "Structured Questionnaires") OR (MM "Surveys+") |
| S37 | TI ("self help" or "self efficacy") |
| S36 | (MM "Support Groups") |
| S35 | (MM "Self-Efficacy") |
| S34 | (MM "Self Care") |
| S33 | (MM "Support, Psychosocial+") |
| S32 | (MM "Cognitive Therapy+") OR (MM "Behavior Therapy+") |
| S31 | (MM "Physician's Role") |
| S30 | (MM "Health Personnel+") |
| S29 | (MH "Physician Assistants") |
| S28 | (MM "Nurses+") |
| S27 | (MM "Counseling+") |
| S26 | ( (MH "Community Health Nursing") OR (MH "Preventive Health Care") |
| S25 | (MH "Community-Institutional Relations") |
| S24 | (MH "Community Health Workers") |
| S23 | (MM "Health Knowledge") |
| S22 | (MM "Attitude to Health+") |
| S21 | (MM "Health Education/MT") |
| S20 | (MH "Patient Education (Iowa NIC)+") |
| S19 | (MM "Patient Education+") |
| S18 | (MH "Patient Navigation") |
| S17 | AB intervention* N6 (clinician* or collaborat* or community or complex or DESIGN* or doctor* or physician* or practitioner* or educational or financial or GP or general practice* or hospital* or impact* or improv* or individuali?e* or individuali?ing or interdisciplin* or multicomponent or multi-component or multidisciplin* or multi-disciplin* or multifacet* or multi-facet* or multimodal* or multi-modal* or combined or personali?e* or personali?ing or prescrib* or primary care or professional* or provider* or team* or usual care or cognitive or behavioral) |
| S16 | TI intervention* |
| S15 | S4 AND S14 |
| S14 | S5 OR S6 OR S7 OR S8 OR S9 OR S10 OR S11 OR S12 OR S13 |
| S13 | TI ( (cervical or vaginal) W2 (smear or smears) ) OR AB ( (cervical or vaginal) W2 (smear or smears) ) |
| S12 | TI "human papilloma virus*" N2 test* OR AB "human papilloma virus*" N2 test* |
| S11 | TI Human Papillomavirus test* OR AB Human Papillomavirus test* |
| S10 | TI ( HPV N3 (test or tests or testing) ) OR AB ( HPV N3 (test or tests or testing) ) |
| S9 | TI ( (pap or Papanicolaou) N2 (smear or smears or test) ) OR AB ( (pap or Papanicolaou) N2 (smear or smears or test) ) |
| S8 | (MH "Cervical Smears+") |
| S7 | TI ( screening or detection ) OR AB ( screening ) |
| S6 | (MH "Early Detection of Cancer") |
| S5 | (MM "Cancer Screening") |
| S4 | S1 OR S2 OR S3 |
| S3 | TI ( (cervix or cervical) N3 (neoplasm* or cancer or carcinoma* or precancer* or premalignant or dysplasia or Neoplasia) ) OR AB ( (cervix or cervical) N3 (neoplasm* or cancer or carcinoma* or precancer* or premalignant or dysplasia or Neoplasia) ) |
| S2 | (MH "Cervix Neoplasms+") |
| S1 | (MH "Cervical Intraepithelial Neoplasia+") |

Supplementary Table S5. Cochrane Library search strategy

| #1 | MeSH descriptor: [Uterine Cervical Neoplasms] explode all trees |
| --- | --- |
| #2 | MeSH descriptor: [Cervical Intraepithelial Neoplasia] explode all trees |
| #3 | MeSH descriptor: [Uterine Cervical Dysplasia] explode all trees |
| #4 | ((cervix or cervical) NEAR/3 (neoplasm* or cancer or carcinoma* or precancer or precancerous or premalignant or dysplasia or neoplasia)):ti,ab,kw (Word variations have been searched) |
| #5 | {OR #1-#4} with Cochrane Library publication date Between Jan 2000 and Dec 2020 |
| #6 | MeSH descriptor: [Mass Screening] this term only |
| #7 | MeSH descriptor: [Early Detection of Cancer] this term only |
| #8 | (screening or detection):ti,ab,kw (Word variations have been searched) |
| #9 | MeSH descriptor: [Papanicolaou Test] this term only |
| #10 | ((HPV NEAR/3 (test or tests or testing))):ti,ab,kw (Word variations have been searched) |
| #11 | (Human Papillomavirus test*):ti,ab,kw (Word variations have been searched) |
| #12 | (("human papilloma virus*" NEAR/2 test*)):ti,ab,kw (Word variations have been searched) |
| #13 | (((pap or papanicolaou) NEAR (smear or smears or test))):ti,ab,kw (Word variations have been searched) |
| #14 | MeSH descriptor: [Vaginal Smears] explode all trees |
| #15 | (((cervical or vaginal) NEAR (smear or smears))):ti,ab,kw (Word variations have been searched) |
| #16 | {OR #6-#15} |
| #17 | #5 AND #16 |
| #18 | (intervention*):ti,ab,kw (Word variations have been searched) |
| #19 | MeSH descriptor: [Patient Navigation] this term only |
| #20 | MeSH descriptor: [Patient Education as Topic] this term only |
| #21 | MeSH descriptor: [Health Education] this term only |
| #22 | MeSH descriptor: [Health Promotion] this term only |
| #23 | MeSH descriptor: [Health Knowledge, Attitudes, Practice] explode all trees |
| #24 | MeSH descriptor: [Community Health Workers] this term only |
| #25 | MeSH descriptor: [Community-Institutional Relations] this term only |
| #26 | MeSH descriptor: [Community Health Services] this term only |
| #27 | MeSH descriptor: [Counseling] explode all trees |
| #28 | MeSH descriptor: [Nurses] explode all trees |
| #29 | MeSH descriptor: [Physician Assistants] this term only |
| #30 | MeSH descriptor: [Health Personnel] this term only |
| #31 | MeSH descriptor: [Allied Health Personnel] this term only |
| #32 | MeSH descriptor: [Health Educators] this term only |
| #33 | MeSH descriptor: [Physicians] this term only |
| #34 | MeSH descriptor: [Physician's Role] this term only |
| #35 | MeSH descriptor: [Cognitive Behavioral Therapy] explode all trees |
| #36 | MeSH descriptor: [Social Support] explode all trees |
| #37 | MeSH descriptor: [Self Care] this term only |
| #38 | MeSH descriptor: [Self Efficacy] this term only |
| #39 | MeSH descriptor: [Self-Help Groups] this term only |
| #40 | (("self help" or "self efficacy")):ti (Word variations have been searched) |
| #41 | MeSH descriptor: [Surveys and Questionnaires] this term only |
| #42 | ((education* or training or navigator* or "patient navigation" or promotoras or "community health workers" or "lay health workers" or "community outreach" or "community clinic" or "federal* qualified health center*")):ti (Word variations have been searched) |
| #43 | {OR #18-#42} |
| #44 | #17 AND #43 |
